# Unraveling the relationships between midge abundance and incidence, microbial communities, and soil and water properties in a protected natural tallgrass prairie

**DOI:** 10.1186/s13071-025-06780-5

**Published:** 2025-04-19

**Authors:** Saraswoti Neupane, Travis Davis, Cassandra Olds, Dana Nayduch, Bethany L. McGregor

**Affiliations:** 1 Center for Grain and Animal Health Research, Arthropod-Borne Animal Diseases Research Unit, USDA-ARS, Manhattan, KS 66502 USA; 2 Department of Entomology, Kansas State University, Manhattan, KS 66506 USA

**Keywords:** Biting midge, *Culicoides*, Bacteria, Protist, Microbial community, Soil, Water

## Abstract

**Background:**

Biting midges (*Culicoides* spp.) are small blood-feeding flies (Diptera: Ceratopogonidae) that transmit numerous pathogens that impact animal and human health. The larvae of several *Culicoides* spp., including vectors, are often found in organically enriched, moist soil habitats. However, the influence of biotic (e.g., cohabiting fauna, potential prey taxa) and abiotic factors (e.g., soil or water properties, time) on abundance and incidence of larval *Culicoides* in natural habitats is not well understood. This study evaluated the relationships between bacterial and protistan communities, soil and water physicochemical properties, and the abundance and incidence of *Culicoides* species in larval habitats at the Konza Prairie Biological Station in Kansas.

**Methods:**

Soil and water samples were collected monthly from March 2021 to February 2022 from four midge larval habitat sites, including three grazed (low-production cattle-grazed (LPCG), high-production cattle-grazed (HPCG), and bison-grazed sites) and one formally ungrazed (i.e., no managed large mammals) site. Midge incidence and abundance were evaluated using emergence assays, which assessed the number of adults emerging from collected soil samples, and bacterial and protistan communities in these samples were characterized through amplicon sequencing of the 16S and 18S rRNA genes. Physicochemical properties of water and soil were also analyzed.

**Results:**

Irrespective of site, the highest midge abundance was reported in warmer months between March and September, except June. Moreover, the greatest midge abundance, incidence, and prevalence occurred at the HPCG and bison-grazed sites, which had a persistent water source. Specific lineages of bacterial and protistan communities, soil texture, organic matter, and total dissolved solids in water samples were directly associated with the abundance of *Culicoides* spp. that emerged from soil samples.

**Conclusions:**

Both biotic (bacterial and protistan communities, presence of host animals), and abiotic (soil and water properties, season) factors affected the abundance and incidence of *Culicoides* spp. in natural habitats. The results presented in this study expand our understanding of the ecological and environmental factors influencing larval ecology of biting midges in natural developmental substrates. These insights have important implications for identifying potential developmental sites, which can be used for targeted management of *Culicoides*.

**Graphical Abstract:**

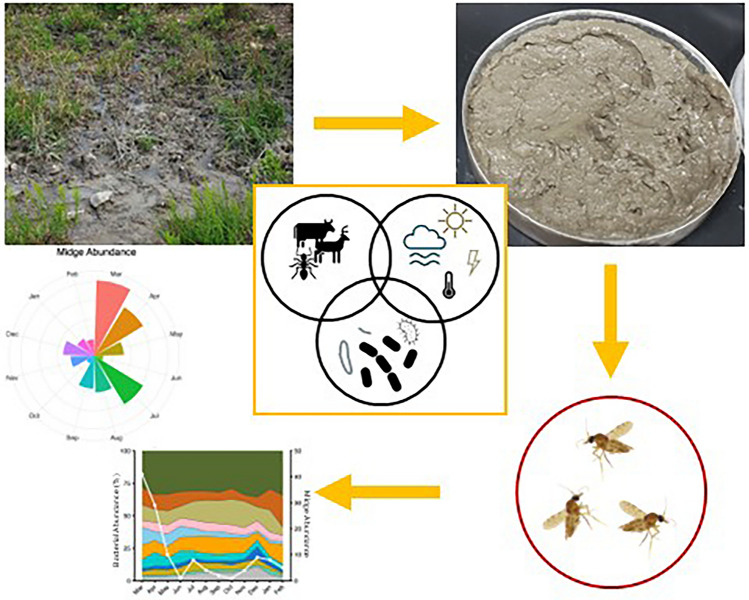

**Supplementary Information:**

The online version contains supplementary material available at 10.1186/s13071-025-06780-5.

## Background

Biting midges (*Culicoides* spp.) are small hematophagous flies that can cause annoyance to humans and animals. Adult females of most *Culicoides* spp. feed on a diverse range of hosts and are considered important vectors of numerous pathogens, including viruses, filarial worms, and protozoa, which can affect the health of domestic and wild animals, as well as humans. In North America, *Culicoides sonorensis* is one of the only confirmed vectors of bluetongue virus (BTV), epizootic hemorrhagic disease virus (EHDV), and vesicular stomatitis virus (VSV) in wild and agricultural animals, although several additional putative vectors probably contribute to disease spread, such as *C. stellifer, C. venustus,* and *C. variipennis* (reviewed in [1]). These diseases can result in substantial economic losses for farmers and ecological impacts to wildlife populations due to animal morbidity and mortality [2–5].

The larvae of numerous *Culicoides* species develop in habitats characterized by organic-matter- and microbe-rich moist soil, including semiaquatic environments such as streams, springs, wetlands, and animal waste ponds [6, 7]. Confirmed and suspected vector species of the genus *Culicoides* have been reported from areas with large numbers of potential host animals such as deer and livestock [8–12]. The presence of *Culicoides* in proximity to hosts and their ability to transmit a variety of microbial pathogens make them a major threat to the health and productivity of both wild and agricultural animals. However, our understanding of suitable natural habitat characteristics for the development of most *Culicoides* species is still limited.

Numerous abiotic and biotic factors influence the *Culicoides* community present in natural larval habitats. For instance, soil properties such as organic matter, moisture, and phosphorus have been found to impact the abundance of several *Culicoides* spp., including vector species [13–17]. In addition to soil properties, seasonality [18] and the presence of abundant host species, such as livestock [11, 17, 18], influenced the abundance and prevalence of *Culicoides* spp. Microbial communities in the soil can directly influence the prevalence of *Culicoides* spp. that feed on live microbes, which are essential for their successful development and growth [16, 19]. Although microbes play a crucial role in the survival of *Culicoides*, significant knowledge gaps remain on the influence of microbial communities, soil chemistry, and water properties on *Culicoides* spp. in their natural habitats.

Surveying the bacterial and protistan communities and the soil and water properties of larval midge habitats will improve our understanding of the ecological factors that support the survival and breeding of *Culicoides* spp., improving our ability to develop targeted control strategies for midge larvae. Thus, we analyzed the characteristics of *Culicoides* larval habitats that include soil properties, water properties, and soil microbial (bacterial and protistan) communities, and determined how these factors correlated and potentially influenced the abundance and incidence of *Culicoides* spp. in larval habitats in a natural protected area.

## Methods

Four sites were chosen within the Konza Prairie Biological Station (KPBS), a protected natural area for long-term ecological research located south of Manhattan, Kansas (39° 05’N, 96° 35’W). The KBPS consists of rolling hills of tallgrass prairie grassland with managed herds of bison (*Bison bison*) and cattle (*Bos taurus*) and natural water sources such as streams, springs, and ponds. Wild populations of white-tailed deer (*Odocoileus virginianus*) and elk (*Cervus canadensis*) are also present and have access to the entire area of KPBS. Two of the sites were located in cattle grazed pastures, including a low-production cattle-grazed (LPCG) site that has comparatively dry soil and has historically produced few midges, and a high-production cattle-grazed (HPCG) site that has been found to produce large populations of midges in preliminary studies. The remaining two sites were located on a bison-grazed pasture and in a formally ungrazed pasture where the only large herbivores present were wild cervids. In the bison-grazed site, animals were maintained year round, while in the cattle grazed sites, animals were removed during winter (November–April). All selected larval habitat sites were situated near natural springs.

### Soil and water sampling

From each site, soil and water samples were collected concomitantly once a month from March 2021 to February 2022 (details in Table S1). Soil samples were collected as described previously [17]. Briefly, a composite soil sample of ~1.0 kg was collected from 0 to 5 cm depth from at least ten locations near the soil–water interface around the larval habitat using a garden trowel. From the composite sample, three aliquots of ~1.0 g soil were placed into 1.5 ml Eppendorf tubes for molecular analysis. All soil samples were transported to the laboratory where approximately 400 g of soil was separated for midge emergence assays (described below). The remaining > 500 g of soil was stored at −80 °C until it was used for soil physicochemical analyses. When water was present, approximately 250 ml water were collected into a 250 ml HDPE bottle (Environmental Express, Charleston, SC, USA) using a turkey baster, with care to avoid disturbing the underlying substrate. Water samples were transported to the laboratory and stored at −80 °C until further analyses.

### Midge emergence assays

Midge emergence assays were performed as described previously [17]. Briefly, 200 g of fresh soil were placed onto Petri dishes in duplicate and incubated at 27 °C, ~80% humidity, and 12:12 (light:dark) photoperiod for up to 6 weeks in a controlled environmental chamber (Model 136VL, Percival Scientific Inc, Perry, IA, USA). Emerged adult midges were collected three times a week using a battery-powered aspirator. Adult midges were identified to species using morphological keys and identification aids as described previously [7, 20, 21].

### Soil and water analyses

Approximately 500 g of soil and 250 ml of water from each site were sent to the Soil Testing Lab, Department of Agronomy, Kansas State University (https://www.agronomy.k-state.edu/services/soiltesting/) to analyze the following soil properties: organic matter (OM), total carbon (TC), total nitrogen (TN), phosphorus (P), potassium (K), calcium (Ca), magnesium (Mg), chloride (Cl), copper (Cu), iron (Fe), manganese (Mn), zinc (Zn), soil texture (sand, silt, and clay), electrical conductivity (EC), soil pH, and water properties: total phosphorus (P), total nitrogen (TN), chloride (Cl), total suspended solids (TSS), total dissolved solids (TDS), electrical conductivity (EC), and pH. Soil pH, OM, TC, TN, and P were analyzed for each sample (one sample/month for a year) while additional soil properties (K, Ca, Mg, Cl, Cu, Fe, Mn, Zn, texture, and EC) were analyzed at least three times a year (Additional file: Table S1), assuming soil texture and quantity of some soil nutrients may not change dramatically within 1–4 months. Similarly, water TN, TP, and Cl were analyzed for all collected samples, while TSS, TDS, EC, and pH were analyzed at least three times a year. Due to a lack of water at the LPCG site in most months, water analyses were not performed (Additional file: Table S1). Additionally, due to a lack of water during March in the HPCG site, this sampling month was not considered for further analysis.

### DNA extraction, library preparation, sequencing, and analysis

DNA was extracted from 0.25 g of soil using the MagAttract^®^ Power Soil^®^ DNA KF Kit (384; Qiagen, Hilden, Germany) following manufacturer’s instructions. Genomic DNA was also extracted from positive (a laboratory culture of bacterium, *Escherichia coli*, and pure culture of a *Penicillium* sp.) and negative (nuclease free water) control samples. The quality and quantity of DNA was measured using a NanoDrop^™^ 8000 spectrophotometer (Thermo Fisher Scientific Inc., Waltham, MA, USA), then samples were stored at −20 °C until further processing.

High throughput amplicon sequencing of the V3–V4 region of the 16S rRNA gene and V4 region of the 18S rRNA gene was performed to characterize bacterial and protistan communities of soil, respectively. Library preparation and sequencing were performed at the Genome Sequencing Core, University of Kansas, Lawrence, Kansas as described previously [17]. The amplicon sequence data were analyzed in Quantitative Insights Into Microbial Ecology (QIIME2, v2023.5, [22]) as described previously [23] except for taxonomy, and reference datasets used were SILVA (v138.1, [24]) and protist ribosomal reference database (PR^2^, v5.0.1, [25]) for 16S and 18S rRNA amplicons, respectively. The relative abundance of bacterial and protistan phyla were obtained and were used for further statistical analyses.

All statistical analyses were performed in the R statistical program (v4.3.1) with packages stats (v4.3.1, [26]), *lme4* (1.1–34, [27]), *lmerTest* (v3.1–3, [28]), *emmeans* (v1.8.7), *Hmisc* (v5.1–0), *ggplot2* (v3.4.3, [29]), and *dplyr* (v1.1.3). Total abundance of *Culicoides* (count data) did not meet the normal distribution and assumption of equal variance (Shapiro–Wilk normality test for normal distribution *P* > 0.05; Levene’s test for homogeneity of variance *P* > 0.05). Therefore, a non-parametric Kruskal–Wallis test was performed to evaluate the effect of site on midge abundance. The model consisted of total abundance of *Culicoides* as the response variable and site as the predictor. A post hoc pairwise Wilcoxon rank sum test was applied to examine the significant difference of mean abundance between sites. Monthly mean relative abundance of bacterial and protistan communities at phylum level within a site were calculated and visualized in area plots and used for further analyses. Further, all samples were categorized into two sample groups depending on the incidence (presence or absence) of *Culicoides*. We then evaluated the influence of site and sample groups and their interactions on the relative abundance of identified bacterial and protistan phyla. A linear mixed effect model was performed with the abundance of phyla as a response variable, and site, sample groups (i.e., sample with and without *Culicoides*), and their interaction as predictor variables, and with sampling month nested to site as random effects (to account for pseudo-replication caused by technical replicate (three samples/month for microbial analysis versus one sample/month for midge abundance)). The model was summarized, the estimated marginal means (EMM) were calculated for each group within a site, and a post hoc Tukey test was performed to compare the group EMMs. The relationships between abundance of *Culicoides* spp. or total abundance, soil properties, texture, electrical conductivity, water properties, water electrical conductivity, and abundance of bacterial phyla and protistan phyla were determined by Spearman’s correlation. Before calculating the correlation coefficients, missing values of soil and water properties were replaced or removed, and in some cases log transformed. Missing values due to partial analyses of soil properties in certain sampling months (see details above, Additional file: Table S1) were replaced with means of soil variables for respective sites. Further, if two or more variables were highly correlated (correlation coefficient > 0.90) then only one of the soil characters were selected. For instance, the correlation coefficient was > 0.90 between P and Ca, Mg and Cu, clay or sand and silt; therefore P, Mg, clay, and sand were chosen. Values of soil properties K and Mg were log transformed. All samples from the LPCG site were removed from the water properties dataset due to lack of water during most of the sampling months (10 out of 12). Therefore, the influence of water properties was evaluated in samples from three sites only (HPCG, bison-grazed, and ungrazed). Spearman correlation coefficients ≥ 0.5 or ≤ −0.5 with *P* < 0.01 were considered highly correlated, ≥ 0.4 or ≤ −0.4 with *P* < 0.01 were considered moderately correlated and ≥ 0.3 or ≤ −0.3 with *P* < 0.01 were considered weakly correlated. All other statistical tests with *P* < 0.05 were considered statistically significant.

## Results

### Abundance/prevalence of *Culicoides* and relationship with soil and water properties

**Fig. 1:**
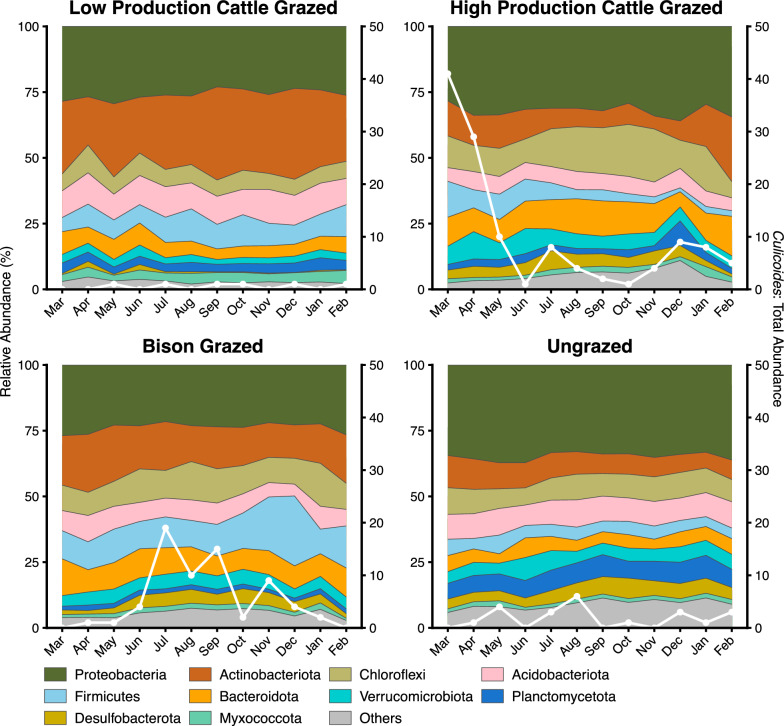
Bacterial communities and abundance of midges in larval habitat soil samples collected from March 2021 to February 2022. Area plots depict mean relative abundances (*n* = 3 soil samples per month/site) of major bacterial phyla. Overlayed lines correspond to secondary vertical axes and represent the number of midges that emerged from soil samples within that month and site

Seven species of *Culicoides*, including *C. crepuscularis*, *C. haematopotus*, *C. stellifer*, *C. sonorensis*, *C. variipennis*, *C. venustus*, and *C. bergi*, were identified from soil samples, with a total abundance of 217 *Culicoides* individuals (Table 1). Although *C. variipennis* (prevalence: 27.1% of all soil samples) was the most abundant, *C. crepuscularis* was the most prevalent species (33.3% of all soil samples). The total number of *Culicoides* varied among sampling months within a site (Fig. 1). The total number of *Culicoides* was also affected by site (Chi square = 19.22, *df* = 3, *P* = 0.0002), where the HPCG site harbored a significantly higher number of mean *Culicoides* compared with ungrazed (*P* = 0.009) and LPCG (*P* = 0.0006) sites.

**Table 1:** Total abundance of *Culicoides* spp. that emerged from soil samples collected from four sites within the Konza Prairie Biological Station

*Culicoides* spp.	Low-production cattle-grazed	High-production cattle-grazed	Bison-grazed	Ungrazed	Total
*C. crepuscularis*	2	18	11	4	35
*C. haematopotus*	2	4	36	1	43
*C. stellifer*	2	6	0	15	23
*C. sonorensis*	0	7	0	0	7
*C. variipennis*	0	78	19	0	97
*C. venustus*	0	2	1	2	5
*C. bergi*	0	7	0	0	7

Various correlations between the abundance of *Culicoides* spp. or total abundance of *Culicoides*, *Culicoides* incidence and soil or water properties were detected (Table 2). For instance, total abundance of *Culicoides* showed a strong positive correlation with amount of Na and Fe in the soil sample. However, a moderate negative correlation was observed between the total abundance of *Culicoides* and amount of soil OM and sand, and a moderate positive correlation with amount of water TDS and EC. Abundance of *Culicoides variipennis* was strongly positively correlated with amount of soil Mg, Cu, and clay, while showing a strong negative correlation with the amount of soil sand particles and water pH. Abundance of other *Culicoides* spp., such as *C. stellifer*, had a moderate positive correlation with the amount of soil TC and EC. Weak negative correlations were observed between abundance of *C. stellifer* and amount of soil P, K, Ca, Mg, and Cu. Abundance of *C. sonorensis* had a weak positive correlation with the amount of soil silt content (Table 2).

**Table 2:** Correlations between number of *Culicoides* in soil samples and environmental factors (soil and water properties)

Soil and water properties^a^	*Culicoides crepuscularis*	*Culicoides haematopotus*	*Culicoides stellifer*	*Culicoides sonorensis*	*Culicoides variipennis*	*Culicoides venustus*	*Culicoides bergi*	*Culicoides*: total abundance	*Culicoides*: incidence
Soil (*n* = 48)									
pH	0.15	0.08	0.34^*^	0.00	−0.06	−0.12	0.18	0.24	0.23
OM	−0.21	−0.13	−0.19	−0.18	−0.18	−0.09	−0.01	−0.43^**^	−0.26
TN	−0.28	−0.21	−0.15	0.07	−0.05	−0.09	−0.05	−0.30	−0.16
TC	−0.15	−0.33	0.43^**^	0.06	−0.25	0.14	0.13	−0.09	−0.07
P	−0.14	0.07	−0.36^*^	−0.13	−0.07	−0.28	−0.29	−0.34^*^	−0.24
Cl	−0.22	−0.30	0.22	0.23	−0.02	0.32	0.11	0.05	0.06
K	−0.04	0.08	−0.38^*^	−0.08	0.04	−0.28	−0.17	−0.24	−0.17
Ca	−0.09	0.07	−0.37^*^	−0.15	0.02	−0.15	−0.13	−0.28	−0.12
Mg	0.33	0.32	−0.36^*^	0.14	0.53^***^	−0.06	0.05	0.39^*^	0.24
Na	0.37^*^	0.33	−0.08	0.13	0.45^**^	0.18	0.11	0.51^***^	0.34^*^
Cu	0.31	0.38^*^	−0.36^*^	0.12	0.52^***^	−0.01	0.02	0.43^**^	0.28
Zn	−0.03	0.00	−0.12	0.10	0.01	−0.15	0.15	−0.17	−0.12
Fe	0.39^*^	0.21	−0.02	0.26	0.41^**^	0.25	0.21	0.58^***^	0.37^*^
Mn	0.43^**^	0.16	−0.06	0.15	0.24	0.20	0.03	0.34^*^	0.19
EC	−0.08	−0.25	0.45^**^	0.21	−0.18	0.23	0.16	0.06	0.05
Sand	−0.30	−0.26	0.13	−0.33	−0.52^***^	−0.03	−0.31	−0.49^**^	−0.30
Silt	0.16	0.06	−0.05	0.35^*^	0.29	−0.02	0.36^*^	0.26	0.14
Clay	0.29	0.36^*^	−0.32	0.15	0.51^***^	−0.05	0.06	0.38^*^	0.23
Water (*n* = 35)									
TN	0.08	0.17	−0.40^**^	−0.10	0.01	−0.04	−0.32	−0.11	−0.29
TP	−0.12	0.04	−0.02	−0.08	−0.27	0.01	−0.24	−0.20	−0.19
Cl	0.14	0.33	−0.42^**^	−0.05	0.30	−0.26	−0.15	0.21	−0.13
TSS	−0.08	−0.02	0.05	0.04	−0.23	−0.20	−0.40^**^	−0.06	−0.13
TDS	0.20	0.15	−0.14	0.28	0.45^**^	−0.10	0.35	0.42^**^	0.27
EC	0.19	0.14	−0.10	0.28	0.45^**^	−0.04	0.35	0.43^**^	0.30
pH	0.00	0.03	0.33	0.01	−0.50^***^	0.13	0.23	−0.15	−0.16

Spearman’s correlation coefficients are shown

^a^Soil organic matter (OM), total carbon (TC), total nitrogen (TN), phosphorus (P), potassium (K), calcium (Ca), magnesium (Mg), chloride (Cl), copper (Cu), iron (Fe), manganese (Mn), zinc (Zn), soil texture (sand, silt, and clay), and water phosphorus (P), water nitrogen (TN), water chloride (Cl), total suspended solids (TSS), total dissolved solids (TDS), water electrical conductivity (EC), and water pH (pH)

^*^Weak correlation: coefficient ≥ 0.3 or ≤ −0.3 and *P* ≤ 0.01

^**^Moderate correlation: coefficient ≥ 0.4 or ≤ −0.4 and *P* ≤ 0.01

^***^Strong correlation: coefficient ≥ 0.5 or ≤ −0.5 and *P* ≤ 0.01

Overall, in each site, soil and water properties ranged widely among sampling months (Additional file: Table S2). The soil composition at each site had variable particle sizes from moderately fine clay loam (LPCG), silty clay loam (HPCG), silty clay (bison-grazed) to moderately course loam (ungrazed). All these soil types had moderate levels of clay content (< 41%; Additional file: Table S2).

### Bacterial and protistan community composition and their relationships with abundance of *Culicoides* spp.

**Fig. 2:**
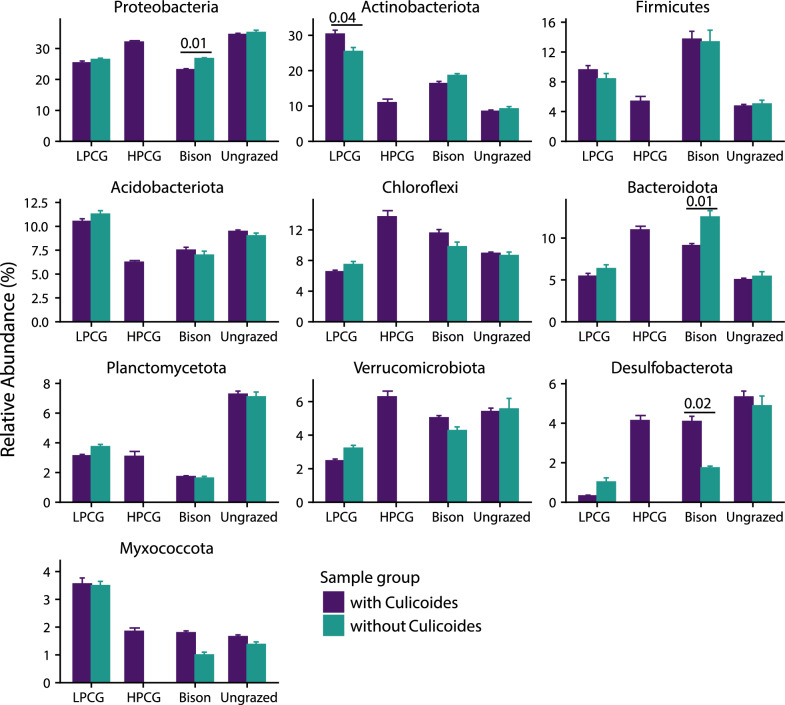
Effect of *Culicoides* presence on relative abundances of major bacterial phyla in soil samples from midge larval habitats. Estimated marginal means of abundance of bacterial phyla between sample groups within a site (low-production cattle-grazed site, LPCG: *n* = 18 per group; high-production cattle-grazed site, HPCG: *n* = 36 with *Culicoides* group, bison-grazed site, bison: *n* = 30 with and 6 without *Culicoides* groups, ungrazed: *n* = 24 with and 12 without *Culicoides* groups) are shown and error bars are standard errors of mean. *P*-values between bars indicate the significant differences in mean relative abundance of bacterial phyla between sample groups (with and without *Culicoides*) within a site (*P* < 0.05)

Irrespective of site and sampling month, the most dominant bacterial phyla consisted of Proteobacteria (relative abundance range 19.66–38.27%), Actinobacteriota (4.29–38.42%), Chloroflexi (1.29–23.12%), Acidobacteriota (1.85–13.81%), Firmicutes (1.08–31.61%), Bacteroidota (2.53–17.89%), Verrucomycrobiota (1.5–12.05%), Planctomycetota (0.96–9.77%), Desulfobacterota (0.01–8.37%), and Myxococcota (0.53–5.42%). Abundances of these phyla varied between sampling months within a site (Fig. 1). Samples with *Culicoides* (sample groups) significantly affected the abundances of Proteobacteria (*F*_(1, 41)_ = 6.34, *P* = 0.02) and Bacteroidota (*F*_(1, 41)_ = 5.89, *P* = 0.02). However, the bison-grazed site samples with *Culicoides* had significantly lower relative abundance of phylum Proteobacteria (*t* = −2.51, *P* = 0.02) and Bacteroidota (*t* = −2.61, *P* = 0.01) compared with samples without *Culicoides* (Fig. 2), while the relative abundance of Desulfobacterota was significantly higher (*t* = 2.38, *P* = 0.02) in samples with *Culicoides* in the bison-grazed site. Similarly, the relative abundance of Actinobacteriota was significantly higher (*t* = 2.14, *P* = 0.04) in samples with *Culicoides* than in samples without *Culicoides* in the LPCG site (Fig. 2).

**Fig. 3:**
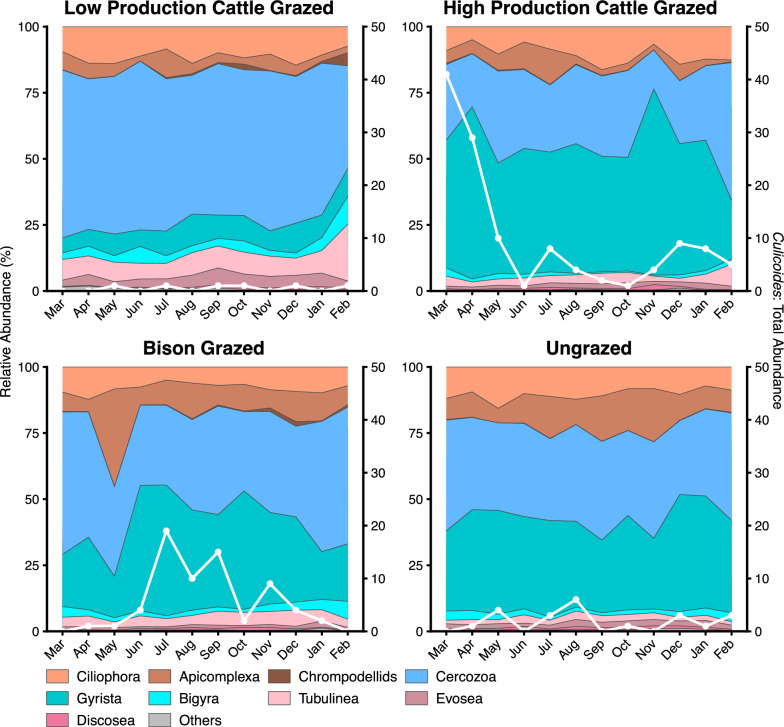
Protistan communities and abundance of midges in larval habitat soil samples collected from March 2021 to February 2022. Area plots depict mean relative abundances (*n* = 3 soil samples per month/site) of major bacterial phyla. Overlayed lines correspond to secondary vertical axes and represent the number of midges that emerged from soil samples within that month and site

**Fig. 4:**
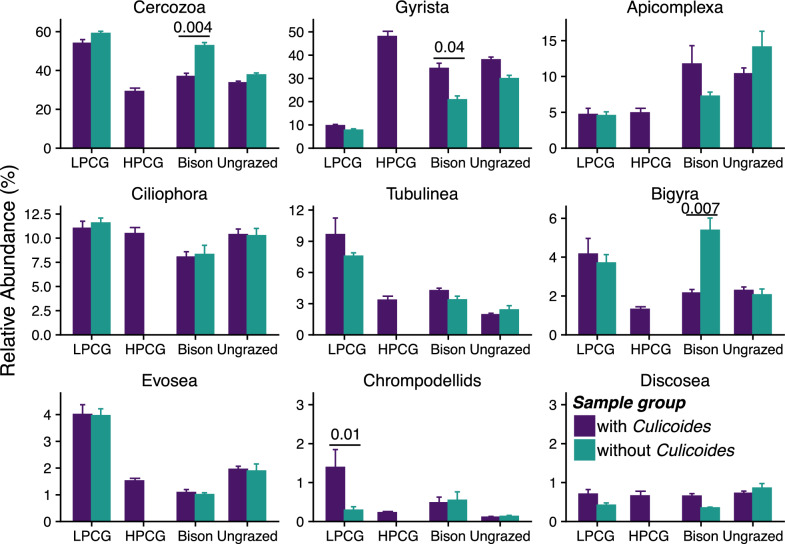
Effect of *Culicoides* presence on relative abundances of major protistan phyla in soil samples from midge larval habitats. Estimated marginal means of abundance of protistan phyla between sample groups within a site (low-production cattle-grazed site, LPCG: *n* = 18 per group, high-production cattle-grazed site, HPCG: *n* = 36 with *Culicoides* group, bison-grazed site, bison: *n* = 30 with and 6 without *Culicoides* groups, ungrazed: *n* = 24 with and 12 without *Culicoides* groups) are shown and error bars are standard errors of the mean. *P*-values between bars indicate the significant differences in mean relative abundance of protistan phyla between sample groups (with and without *Culicoides*) within a site (*P* < 0.05)

Irrespective of site and sampling month, protistan communities were dominated by Ciliophora (relative abundance range 2.89–17.9%), Apicomplexa (0.06–80.51%), Cercozoa (10.3–66.88%), Gyrista (5.01–76.87%), Bigyra (0.35–13.82%), Chrompodellids (0–6.35%), Tubulinea (0.59–34.07%), Evosea (0.19–7.45%), and Discosea (0.03–4.3%). Although the total abundance of *Culicoides* in each month within a site varied (Fig. 3), the site-by-sample groups (sample groups: samples with and without *Culicoides*) interaction significantly influenced the abundances of Bigyra (*F*_(2, 41)_ = 3.73, *P* = 0.03) while sample groups (samples with and without *Culicoides*) significantly affected the abundances of Cercozoa (*F*_(1, 41)_ = 10.65, *P* = 0.002), and Gyrista (*F*_(1, 41)_ = 5.95, *P* = 0.02). In the bison-grazed site, samples with *Culicoides* had significantly lower relative abundances of the phyla Cercozoa (*t* = −3.05, *P* = 0.004) and Bigyra (*t* = −2.80, *P* = 0.007) compared with samples without *Culicoides* (Fig. 4), while the relative abundance of Gyrista was significantly greater in samples with *Culicoides* (*t* = 2.07, *P* = 0.04).

Although various levels of strong, moderate, and weak correlations were observed between soil or water properties and relative abundances of bacterial and protistan phyla, only a few strong or moderate correlations were detected between total abundance of *Culicoides* or abundances and incidence of *Culicoides* spp. and the relative abundances of bacterial and protistan phyla (Table 3). Abundance of *C. variipennis* was strongly negatively correlated with abundances of Acidobacteriota and Planctomycetota, while a moderate positive correlation was found with Chloroflexi and Bacteroidota. A moderate positive correlation was observed between abundance of *C. venustus* and the relative abundance of Desulfobacterota (Table 3). Further, a strong negative correlation was observed between relative abundance of Acidobacteriota and total abundance of *Culicoides*. Strong-to-weak positive correlations were detected between total abundance of *Culicoides* and relative abundances of Chloroflexi, Verrucomicrobiota, Desulfobacterota, and Bacteroidota (Table 3). Similarly, abundance and incidence of Gyrista (Stramenopiles) was strongly positively correlated with total abundance and incidence of *Culicoides*, while Cercozoa (Rhizaria) and Evosea (Amoebozoa) were negatively correlated (Table 3). Similar to total abundance, abundance of *C. crepuscularis, C. sonorensis*, *C. variipennis*, and *C. bergi* were positively correlated with abundance of Gyrista (Stramenopiles) (Table 3).

**Table 3:** Correlations between number of *Culicoides* in soil samples and microbial communities

	*Culicoides crepuscularis*	*Culicoides haematopotus*	*Culicoides stellifer*	*Culicoides sonorensis*	*Culicoides variipennis*	*Culicoides venustus*	*Culicoides bergi*	*Culicoides:* total abundance	*Culicoides:* incidence
Bacteria									
Proteobacteria	−0.12	−0.36^*^	0.35^*^	0.09	−0.17	0.09	0.16	−0.04	−0.09
Actinobacteriota	−0.13	0.05	−0.23	−0.12	−0.05	−0.31	−0.17	−0.29	−0.20
Chloroflexi	0.25	0.29	−0.02	0.23	0.42^**^	0.13	0.29	0.51^***^	0.30
Acidobacteriota	−0.37^*^	−0.09	−0.06	−0.30	−0.60^***^	−0.25	−0.26	−0.57^***^	−0.37^*^
Firmicutes	0.10	0.08	−0.34^*^	0.08	0.19	−0.11	−0.31	0.00	−0.05
Bacteroidota	0.26	0.10	−0.04	0.26	0.48^**^	0.08	0.14	0.34^*^	0.19
Verrucomicrobiota	0.34^*^	0.09	0.13	0.38^*^	0.34^*^	0.28	0.12	0.50^***^	0.36^*^
Planctomycetota	−0.22	−0.28	0.28	−0.13	−0.50^***^	−0.04	0.11	−0.33	−0.23
Desulfobacterota	0.27	0.10	0.16	0.13	0.09	0.41^**^	0.13	0.41^**^	0.25
Myxococcota	0.03	0.08	0.05	−0.12	−0.18	−0.17	0.08	−0.16	−0.02
Protista									
Alveolata	−0.11	−0.10	0.05	−0.15	−0.31	0.15	0.10	−0.18	−0.07
Ciliophora	−0.15	−0.11	0.23	−0.23	−0.27	−0.18	0.17	−0.20	−0.14
Apicomplexa	0.00	0.06	−0.06	0.00	−0.12	0.18	−0.03	−0.03	−0.02
Chrompodellids	0.29	0.21	−0.22	0.07	0.18	−0.16	−0.13	0.12	0.12
Rhizaria	−0.34^*^	−0.10	−0.27	−0.37^*^	−0.32	−0.30	−0.38^*^	−0.63^***^	−0.52^***^
Cercozoa	−0.34^*^	−0.10	−0.27	−0.37^*^	−0.32	−0.30	−0.38^*^	−0.63^***^	−0.52^***^
Stramenopiles	0.41^**^	0.16	0.24	0.37^*^	0.36^*^	0.22	0.34^*^	0.68^***^	0.50^***^
Gyrista	0.41^**^	0.19	0.23	0.34^*^	0.35^*^	0.22	0.35^*^	0.69^***^	0.52^***^
Bigyra	−0.20	−0.11	−0.08	−0.11	−0.20	−0.25	−0.25	−0.42^**^	−0.34^*^
Metamonada	−0.15	0.26	−0.16	0.10	0.17	0.00	0.02	0.09	−0.05
Tubulinea	−0.04	0.02	−0.18	−0.15	−0.01	−0.23	−0.19	−0.27	−0.18
Evosea	−0.37^*^	−0.07	0.09	−0.21	−0.48^**^	−0.10	0.04	−0.48^**^	−0.29
Discosea	0.32	−0.02	−0.03	0.05	0.01	0.31	0.02	0.19	0.18

Spearman’s correlation coefficients are shown

^*^Weak correlation: coefficient ≥ 0.3 or ≤ −0.3 and *P* ≤ 0.01

^**^Moderate correlation: coefficient ≥ 0.4 or ≤ −0.4 and *P* ≤ 0.01

^***^Strong correlation: coefficient ≥ 0.5 or ≤ −0.5 and *P* ≤ 0.01.1

## Discussion

The findings of this study highlight the influence of biotic (bacterial and protistan communities) and abiotic (soil and water properties) factors on the incidence and abundance of larval *Culicoides* spp. in the natural tallgrass prairie ecosystem. To our knowledge, this is the first comprehensive study that evaluated the relationships between *Culicoides* communities, bacterial and protistan communities, and soil and water properties sampled throughout a year in *Culicoides* larval habitats. One of the major findings of this study was that abundances of certain bacterial and protistan phyla and soil and water properties were correlated with both the incidence and abundance of the immature stages of *Culicoides* in the larval habitat, which will influence the composition of the adult midge populations produced from these sites.

The abundance and/or prevalence of *Culicoides* spp. was influenced by a myriad of factors. Distinguishing which factors had the greatest impacts can be challenging because both abiotic (environmental) and biotic (micro- and macro-organisms) factors play a crucial role in the soil ecosystems. Despite sampling similarly from each site, we found a greater abundance and prevalence of *Culicoides* in moist larval habitats that large mammals occupy (HPCG and bison-grazed sites) than in the ungrazed site and the drier LPCG site. This finding is consistent with our prior work that demonstrated a higher prevalence of *Culicoides* in areas inhabited by large grazing mammals than in nongrazed habitats. This indicates that the availability of proximal blood meals for host-seeking adult female midges may drive oviposition, and consequently, larval habitat selection and location [17]. Moreover, the immature stages of several *Culicoides* spp., including confirmed and putative vectors such as *C. sonorensis* and *C. stellifer* [1, 30], have been found to inhabit microbe-rich substrates or semiaquatic habitats with high levels of organic matter, often due to enrichment with animal waste (e.g., near livestock facilities) [13, 31, 32]. These substrates likely provide larval stages of *Culicoides* with abundant microbes for feeding [33, 34]. Although several *Culicoides* species have been found in association with organic matter enriched habitats, our study showed that elevated levels of organic matter in soil negatively impacted the total abundance of *Culicoides.*. The influence of organic matter on larval abundance varies by species [16], presumably due to the broad ecological and behavioral differences among *Culicoides* species .

The abundance of several bacterial phyla (Proteobacteria, Chloroflexi, Bacteroidota, Acidiobacteriota, Planctomycetota, and Verrucomicrobia) was significantly associated with the total abundance of *Culicoides* and/or abundances of individual *Culicoides* spp. Although the observed bacterial phyla have been found in similar protected natural tallgrass prairie soils [17, 35–37], a positive correlation of total abundance of *Culicoides* and Verrucomicrobiota suggested that bacteria in this phylum could have mutualistic relationships with the immature stages of *Culicoides*. For example, Verrucomicrobiota have been shown to degrade phytoplankton into organic matter [38], which is then released into the environment and is eventually available as nutrient resources for numerous organisms, including *Culicoides*. Thus, a greater abundance of Verrucomicrobiota could create favorable conditions for midges, such as *C. crepuscularis*, *C. variipennis*, and *C. sonorensis,*, which seem to prefer semiaquatic, organic-matter-rich larval environments [31, 32, 39, 40].

*Culicoides* larvae nutritionally require microbes for survival and development [34]. The detection of lower abundance, and apparent depletion, of protistan communities including the Cercozoa (Rhizaria), Bigyra (Stramenopiles), and Evosea (Amoebozoa) in the presence of abundant *Culicoides* spp. implies that larvae may also consume these higher trophic level microbes (protists). This finding is not surprising as the immature stages of several *Culicoides* spp. are omnivorous , feeding on not only organic matter (e.g., plant debris), but also on a variety of organisms such as diatoms, nematodes, green algae, bacteria, fungi, and arthropods (reviewed in [40–42]. Moreover, larvae of certain *Culicoides* sp. feeding on protists improved their survival and growth [19], providing direct evidence that *Culicoides* consumed protists. A recent in vitro experiment with another Dipteran species, the house fly, has shown that larval grazing reduced the abundance of Cercozoa in its substrate, possibly due to feeding on these microbes [43]. Similarly, observations of increased abundances of Acidiobacteriota and Planctomycetota in soil associated with lower abundances of total *Culicoides* and *C. variipennis* in our study suggest that *Culicoides* spp. larvae may also consume these bacteria to meet their nutritional requirements, thereby depleting their presence in the soil. Therefore, it is plausible that the omnivorous *Culicoides* larvae could also be feeding on Cercozoa and other protistan groups, leading to the patterns observed in this study.

Soil properties such as soil moisture, pH, OM, P, K, and Zn have also been associated with suitable conditions for survival of various *Culicoides* spp. larvae [13, 14, 16, 44]. However, in the current study, we demonstrated the greater importance of soil texture, along with organic matter and other nutrients (e.g., Na, Mg, Fe, Cu) on abundance of *Culicoides* spp. Since our experimental sites were composed of loam to silty clay loam soil (i.e., high sand and/or silt while moderate clay content), soil clay content had a positive impact while sand content negatively influenced the abundances of total *Culicoides, C. haematopotus*, and *C. variipennis*. This result is in accordance with a study that showed the positive impact of clayey soil on the abundance of *C. imicola* [45]. Soil with a moderate level of clay content could accelerate the organic matter decomposition process, releasing more nutrient resources in the soil [46], which can subsequently enhance the survival, development, and activities of microorganisms [46] and larvae of *Culicoides* spp. In contrast, soil with high sand content creates spaces within the soil matrix, which allows soil better drainage but lowers the capacity of retaining nutrients in the soil, leading to a nutrient poor environment that would be less suitable for growth and development of soil organisms [47], including immature *Culicoides* spp.

Increased substrate pH was considered favorable for larval development of certain *Culicoides* spp. [15, 16]. In contrast, we observed no significant impact of soil pH on the abundance of *Culicoides* spp., which is presumably due to a narrow range of soil pH across sites and months (or individual soil samples). The sites used in this study were adjacent to springs, which could contribute to surface erosion and the deposition of sand and silt particles causing somewhat uniform soil pH across sampling sites (7.7–8.2). In addition, greater amounts of soil Mg and Cu associated with low abundance of *C. stellifer* and high abundance of *C. variipennis* and *Culicoides* (total abundance) suggest that impacts of specific micro- and macro-nutrients on immature stages of *Culicoides* spp. varies species to species and may be location and habitat-type dependent.

The abundance of bacterial and protistan communities in the larval habitats slightly varied over time, suggesting these communities were influenced by seasonal changes in environmental conditions. Lower anticipated microbial activity in winter months (lowest recorded daily mean temperature at a sampling date, 2.58 °C) and higher activity in summer (highest recorded daily mean temperature at a sampling date, 26.64 °C) likely contributed to the observed variation in soil microbial communities. Temporal variation in microbial activity within the soil subsequently impacts soil nutrient availability since some microorganisms degrade complex organic matter to simple sugars and proteins, which can be utilized by cohabiting soil organisms [48], including midges and other fauna. Previous studies have demonstrated seasonal patterns of activities and breeding behavior of different *Culicoides* species [49–52], with overwintering diapause believed to occur in the larval stage [53]. We observed a greater emergence of midges in soil samples collected during warmer months (March–September, except June) compared with winter months despite all samples being reared in incubators set at 27 °C, likely because those substrates were oviposition sites used during the warmer months. Temperature more significantly influences midge emergence from diapause than photoperiod [54]. However, abrupt changes in conditions (i.e., samples collected from conditions with short daylength and cold temperatures in the field transitioning to long daylength, warm temperatures in the incubator) could have affected survival and emergence of diapausing midges collected during the winter.

There were a few limitations to this study that must be considered. Although each soil sample represented a composite sample of at least ten random collections within a site, we only used two replicates of 200 g soil for each midge emergence assay. Most midge species lay eggs in batches, and as a result, the total abundance of potential midges that could emerge from the sample may not have been captured or may have been overrepresented if larvae were clustered within the substrate. In addition, some soil and water properties were evaluated monthly while others were only evaluated every few months, which could have affected our understanding of the associations between these variables and midge larval emergence. In addition, not all biotic or abiotic factors were captured, including determination of other potential food sources (e.g., invertebrates) or minerals/nutrients critical to midge development. Moreover, bacterial and protistan communities were predicted by using V3–V4 and V4 regions of 16S and 18S rRNA gene-based amplicon sequencing methods, respectively, which may not provide full taxonomic resolution of the microbial community. Therefore, future studies should incorporate larger sample sizes, more replication, and more comprehensive sampling approaches along with shotgun metagenome sequencing methods to address these limitations.

## Conclusions

The findings of this study demonstrated that soil and water physicochemical properties (e.g., soil texture, OM, Na, water TDS) and abundances of specific bacterial and protistan communities influenced the abundance and incidence of immature stages of *Culicoides* in the soil. Changes observed in the microbial community, physical environment, and midge abundance patterns throughout the year demonstrate the dynamic nature of these habitats and the need to consider temporal variation when implementing midge surveillance or control strategies. These insights have important implications for understanding the ecology and management of *Culicoides* in natural breeding sites including both wild and animal agricultural settings.

## Supplementary Information

Additional file 1: Table S1. Metadata information for collection sites and samples. Table S2. Mean and standard deviation of soil and water properties in each sampling site collected once a month for 12 months.

## Data Availability

No datasets were generated or analyzed during the current study.

## Abbreviations

BTV: Bluetongue virus
EHDV: Epizootic hemorrhagic disease virus
VSV: Vesicular stomatitis virus
HPCG: High-production cattle-grazed
LPCG: Low-production cattle-grazed
PCR: Polymerase chain reaction
ASV: Amplicon sequence variant
OM: Organic matter
TC: Total carbon
TN: Total nitrogen
P: Phosphorus
K: Potassium
Ca: Calcium
Na: Sodium
Mg: Magnesium
Cl: Chloride
Cu: Copper
Fe: Iron
Mn: Manganese
Zn: Zinc
EC: Electrical conductivity
TDS: Total dissolved solids
TSS: Total suspended solids
