# Supplementary material for: Unraveling the relationships between midge abundance and incidence, microbial communities, and soil and water properties in a protected natural tallgrass prairie
Source: Parasit Vectors. 2025 Apr 19;18:146. doi: 10.1186/s13071-025-06780-5 (PMC12009528; doi:10.1186/s13071-025-06780-5)
Supplement: Supplementary file 1 — Additional file 1: Table S1. Metadata information for collection sites and samples. Table S2. Mean and standard deviation of soil and water properties in each sampling site collected once a month for 12 months. [file 13071_2025_6780_MOESM1_ESM.pdf]

Supplementary Information

Unravelling the Relationships Between Midge Abundance and Incidence, Microbial Communities, and Soil and Water Properties in a Protected Natural Tallgrass Prairie

Saraswoti Neupane[1†*], Travis Davis[2], Cassandra Olds[1], Dana Nayduch[2], Bethany L. McGregor[2*]

[1]Department of Entomology, Kansas State University, Manhattan, KS 66506, USA

[2]USDA-ARS, Center for Grain and Animal Health Research, Arthropod-Borne Animal Diseases Research Unit, Manhattan, KS 66502, USA

[†]Current Address: USDA-ARS, Center for Grain and Animal Health Research, Arthropod-Borne Animal Diseases Research Unit, Manhattan, KS 66502, USA

Table S1. Metadata information for collection sites and samples.

Site	Grazer[a]	Month	Soil Test Profile[b]	Water Test Profile[c*]	Latitude longitude
Low Production Cattle Grazed	Cattle	Jan	pH, OM, TC, TN, P, Cl, K, Ca, Mg, Na, Mn, Cu, Zn, Fe, EC, Sand, Silt, Clay		39.11449 N 96.563530 W
Low Production Cattle Grazed	Cattle	Feb	pH, OM, TC, TN, P		39.11449 N 96.563530 W
Low Production Cattle Grazed	Cattle	Mar	pH, OM, TC, TN, P		39.11449 N 96.563530 W
Low Production Cattle Grazed	Cattle	Apr	pH, OM, TC, TN, P, Cl, K, Ca, Mg, Na, Mn, Cu, Zn, Fe, EC, Sand, Silt, Clay		39.11449 N 96.563530 W
Low Production Cattle Grazed	Cattle	May	pH, OM, TC, TN, P	P, TN, Cl, TSS, TDS, EC, pH	39.11449 N 96.563530 W
Low Production Cattle Grazed	Cattle	Jun	pH, OM, TC, TN, P		39.11449 N 96.563530 W
Low Production Cattle Grazed	Cattle	Jul	pH, OM, TC, TN, P, Cl, K, Ca, Mg, Na, Mn, Cu, Zn, Fe, EC		39.11449 N 96.563530 W
Low Production Cattle Grazed	Cattle	Aug	pH, OM, TC, TN, P		39.11449 N 96.563530 W
Low Production Cattle Grazed	Cattle	Sep	pH, OM, TC, TN, P	P, TN, Cl, TSS, TDS, EC, pH	39.11449 N 96.563530 W
Low Production Cattle Grazed	Cattle	Oct	pH, OM, TC, TN, P, Cl, K, Ca, Mg, Na, Mn, Cu, Zn, Fe, EC, Sand, Silt, Clay		39.11449 N 96.563530 W
Low Production Cattle Grazed	Cattle	Nov	pH, OM, TC, TN, P		39.11449 N 96.563530 W
Low Production Cattle Grazed	Cattle	Dec	pH, OM, TC, TN, P		39.11449 N 96.563530 W
Ungrazed	None	Jan	pH, OM, TC, TN, P, Cl, K, Ca, Mg, Na, Mn, Cu, Zn, Fe, EC, Sand, Silt, Clay	P, TN, Cl, TSS, TDS, EC, pH	39.104277 N 96.59113 W
Ungrazed	None	Feb	pH, OM, TC, TN, P	P, TN, Cl	39.104277 N 96.59113 W
Ungrazed	None	Mar	pH, OM, TC, TN, P	P, TN, Cl	39.104277 N 96.59113 W
Ungrazed	None	Apr	pH, OM, TC, TN, P, Cl, K, Ca, Mg, Na, Mn, Cu, Zn, Fe, EC, Sand, Silt, Clay	P, TN, Cl, TSS, TDS, EC, pH	39.104277 N 96.59113 W
Ungrazed	None	May	pH, OM, TC, TN, P	P, TN, Cl	39.104277 N 96.59113 W
Ungrazed	None	Jun	pH, OM, TC, TN, P	P, TN, Cl	39.104277 N 96.59113 W

Ungrazed	None	Jul	pH, OM, TC, TN, P, Cl, K, Ca, Mg, Na, Mn, Cu, Zn, Fe, EC	P, TN, Cl, TSS, TDS, EC, pH	39.104277 N 96.59113 W
Ungrazed	None	Aug	pH, OM, TC, TN, P	P, TN, Cl	39.104277 N 96.59113 W
Ungrazed	None	Sep	pH, OM, TC, TN, P	P, TN, Cl	39.104277 N 96.59113 W
Ungrazed	None	Oct	pH, OM, TC, TN, P, Cl, K, Ca, Mg, Na, Mn, Cu, Zn, Fe, EC, Sand, Silt, Clay	P, TN, Cl, TSS, TDS, EC, pH	39.104277 N 96.59113 W
Ungrazed	None	Nov	pH, OM, TC, TN, P	P, TN, Cl	39.104277 N 96.59113 W
Ungrazed	None	Dec	pH, OM, TC, TN, P	P, TN, Cl	39.104277 N 96.59113 W
Bison Grazed	Bison	Jan	pH, OM, TC, TN, P, Cl, K, Ca, Mg, Na, Mn, Cu, Zn, Fe, EC, Sand, Silt, Clay	P, TN, Cl, TSS, TDS, EC, pH	39.076411 N 96.568156 W
Bison Grazed	Bison	Feb	pH, OM, TC, TN, P	P, TN, Cl	39.076411 N 96.568156 W
Bison Grazed	Bison	Mar	pH, OM, TC, TN, P, Cl, K, Ca, Mg, Na, Mn, Cu, Zn, Fe, Sand, Silt, Clay	P, TN, Cl, TSS, TDS, EC, pH	39.076411 N 96.568156 W
Bison Grazed	Bison	Apr	pH, OM, TC, TN, P, Cl, K, Ca, Mg, Na, Mn, Cu, Zn, Fe, Sand, Silt, Clay	P, TN, Cl, TSS, TDS, EC, pH	39.076411 N 96.568156 W
Bison Grazed	Bison	May	pH, OM, TC, TN, P, Cl, K, Ca, Mg, Na, Mn, Cu, Zn, Fe, Sand, Silt, Clay	P, TN, Cl, TSS, TDS, EC, pH	39.076411 N 96.568156 W
Bison Grazed	Bison	Jun	pH, OM, TC, TN, P	P, TN, Cl	39.076411 N 96.568156 W
Bison Grazed	Bison	Jul	pH, OM, TC, TN, P, Cl, K, Ca, Mg, Na, Mn, Cu, Zn, Fe, EC, Sand, Silt, Clay	P, TN, Cl, TSS, TDS, EC, pH	39.076411 N 96.568156 W
Bison Grazed	Bison	Aug	pH, OM, TC, TN, P	P, TN, Cl	39.076411 N 96.568156 W
Bison Grazed	Bison	Sep	pH, OM, TC, TN, P	P, TN, Cl	39.076411 N 96.568156 W
Bison Grazed	Bison	Oct	pH, OM, TC, TN, P, Cl, K, Ca, Mg, Na, Mn, Cu, Zn, Fe, EC, Sand, Silt, Clay	P, TN, Cl, TSS, TDS, EC, pH	39.076411 N 96.568156 W
Bison Grazed	Bison	Nov	pH, OM, TC, TN, P	P, TN, Cl	39.076411 N 96.568156 W
Bison Grazed	Bison	Dec	pH, OM, TC, TN, P	P, TN, Cl	39.076411 N 96.568156 W
High Production Cattle Grazed	Cattle	Jan	pH, OM, TC, TN, P, Cl, K, Ca, Mg, Na, Mn, Cu, Zn, Fe, EC, Sand, Silt, Clay	P, TN, Cl, TSS, TDS, EC, pH	39.113127 N 96.559257 W
High Production Cattle Grazed	Cattle	Feb	pH, OM, TC, TN, P	P, TN, Cl	39.113127 N 96.559257 W
High Production Cattle Grazed	Cattle	Mar	pH, OM, TC, TN, P, Cl, K, Ca, Mg, Na, Mn, Cu, Zn, Fe, Sand, Silt, Clay		39.113127 N 96.559257 W
High Production Cattle Grazed	Cattle	Apr	pH, OM, TC, TN, P, Cl, K, Ca, Mg, Na, Mn, Cu, Zn, Fe, Sand, Silt, Clay	P, TN, Cl, TSS, TDS, EC, pH	39.113127 N 96.559257 W

High Production Cattle Grazed	Cattle	May	pH, OM, TC, TN, P, Cl, K, Ca, Mg, Na, Mn, Cu, Zn, Fe, Sand, Silt, Clay	P, TN, Cl, TSS, TDS, EC, pH	39.113127 N 96.559257 W
High Production Cattle Grazed	Cattle	Jun	pH, OM, TC, TN, P	P, TN, Cl	39.113127 N 96.559257 W
High Production Cattle Grazed	Cattle	Jul	pH, OM, TC, TN, P, Cl, K, Ca, Mg, Na, Mn, Cu, Zn, Fe, EC, Sand, Silt, Clay	P, TN, Cl, TSS, TDS, EC, pH	39.113127 N 96.559257 W
High Production Cattle Grazed	Cattle	Aug	pH, OM, TC, TN, P	P, TN, Cl	39.113127 N 96.559257 W
High Production Cattle Grazed	Cattle	Sep	pH, OM, TC, TN, P	P, TN, Cl	39.113127 N 96.559257 W
High Production Cattle Grazed	Cattle	Oct	pH, OM, TC, TN, P, Cl, K, Ca, Mg, Na, Mn, Cu, Zn, Fe, EC, Sand, Silt, Clay	P, TN, Cl, TSS, TDS, EC, pH	39.113127 N 96.559257 W
High Production Cattle Grazed	Cattle	Nov	pH, OM, TC, TN, P	P, TN, Cl	39.113127 N 96.559257 W
High Production Cattle Grazed	Cattle	Dec	pH, OM, TC, TN, P	P, TN, Cl	39.113127 N 96.559257 W

[a]No formal grazer present in the ungrazed site but large number of animals such as white-tailed deer were roamed freely.

[b]Soil full analysis includes soil organic matter (OM), total carbon (TC), total nitrogen (TN), phosphorus (P), potassium (K), calcium (Ca), magnesium (Mg), chloride (Cl), copper (Cu), iron (Fe), manganese (Mn), zinc (Zn), soil texture (sand, silt and clay) while partial analysis includes pH, OM, TC, TN and P.

[*]Empty cells represent no water properties analyzed due to lack of water sample.

[c]Water full analysis includes water phosphorus (P), water nitrogen (TN), water chloride (Cl), total suspended solids (TSS), total dissolved solids (TDS), water electrical conductivity (EC) and water pH (pH) while partial analysis include P, TN and Cl.

Table S2. Mean and standard deviation of soil and water properties in each sampling site collected once a month for 12 months.

	**Low Production Cattle Grazed**	**High Production Cattle Grazed**	**Bison Grazed**	**Ungrazed**
**Soil properties**				
pH	7.82 ± 0.10	7.94 ± 0.09	7.88 ± 0.09	8.03 ± 0.09
Sikora pH	7.47 ± 0.05	7.49 ± 0.02	7.39 ± 0.06	7.61 ± 0.03
Organic matter (g kg^{-1})	51.58 ± 8.05	41.92 ± 5.85	38.25 ± 2.93	36.58 ± 12.13
Total carbon (g kg^{-1})	59.48 ± 7.72	83.30 ± 4.12	28.54 ± 1.95	106.39 ± 5.23
Total nitrogen (g kg^{-1})	2.20 ± 0.23	2.02 ± 0.24	1.70 ± 0.31	1.58 ± 0.36
Phosphorus (mg kg^{-1})	23.67 ± 6.93	1.20 ± 1.46	7.51 ± 1.24	0 ± 0
Chloride (mg kg^{-1})	8.67 ± 2.42	18.93 ± 9.86	10.03 ± 8.41	18.25 ± 16.07
Potassium (mg kg^{-1})	384.50 ± 48.05	194.38 ± 44.00	308.68 ± 54.50	75.25 ± 25.02

Calcium (mg kg^{-1})	5393.00 ± 257.99	4730.92 ± 163.59	4957.73 ± 322.19	4142 ± 157.13
Magnesium (mg kg^{-1})	274.50 ± 29.03	407.24 ± 62.60	510.96 ± 33.47	202.75 ± 9.81
Sodium (mg kg^{-1})	11.25 ± 1.50	20.35 ± 1.64	30.20 ± 4.17	17.25 ± 5.85
Copper (mg kg^{-1})	1.55 ± 0.44	2.02 ± 0.29	2.95 ± 0.26	1.02 ± 0.21
Zink (mg kg^{-1})	1.60 ± 0.65	0.80 ± 0.18	0.67 ± 0.12	0.45 ± 0.13
Iron (mg kg^{-1})	45.75 ± 19.62	89.25 ± 15.62	83.05 ± 18.27	51.50 ± 31.86
Manganese (mg kg^{-1})	15.50 ± 5.26	16.52 ± 4.83	20.07 ± 10.98	13.50 ± 7.55
Sand (%)	20.67 ± 2.31	15.83 ± 1.60	18.00 ± 1.26	48 ± 3.46
Silt (%)	46.33 ± 3.21	49.67 ± 1.51	43.00 ± 1.55	33.67 ± 2.89
Clay (%)	33.00 ± 1.00	34.50 ± 0.84	39.00 ± 2.00	18.33 ± 0.58
Electrical conductivity (mS kg^{-1})	0.76 ± 0.13	1.07 ± 0.26	0.69 ± 0.02	1.26 ± 0.25
**Water properties**				
Total nitrogen (mg L^{-1})	0.50 ± 0.01	0.24 ± 0.14	1.01 ± 1.79	0.50 ± 0.48
Total phosphorus (mg L^{-1})	0.11 ± 0.01	0.02 ± 0.02	0.05 ± 0.05	0.09 ± 0.10
Chloride (mg L^{-1})	5.15 ± 5.23	6.92 ± 3.41	8.55 ± 3.27	4.96 ± 2.69
Total suspended solids (mg L^{-1})	85.00 ± 35.36	95.80 ± 41.98	111.83 ± 54.32	121.25 ± 16.66
Total dissolved solids (mg L^{-1})	229.5 ± 81.32	294.20 ± 17.20	256.05 ± 26.27	221.25 ± 14.61
pH	8.23 ± 0.11	7.92 ± 0.45	7.94 ± 0.39	8.20 ± 0.04
Electrical conductivity (mS cm^{-1})	0.33 ± 0.11	0.42 ± 0.02	0.36 ± 0.04	0.32 ± 0.02
